# Microwave pretreatment of wastewater sludge technology—a scientometric-based review

**DOI:** 10.1007/s11356-024-32931-9

**Published:** 2024-03-27

**Authors:** Yuxuan Li, Luiza C. Campos, Yukun Hu

**Affiliations:** Department of Civil, Environmental & Geomatic Engineering, University College London, London, WC1E 6BT UK

**Keywords:** Microwave pretreatment, Energy consumption, Biogas production, Anaerobic digestion, Scientometric assessment

## Abstract

This manuscript presents a scientometric review of recent advances in microwave pretreatment processes for sewage sludge, systematically identifying existing gaps and prospects. For this purpose, 1763 papers on the application of microwave technology to sludge pretreatment were retrieved from the Web of Science (WoS) using relevant keywords. These publications were then analyzed using diverse scientometric indices. The results show that research in this field encompasses applications based on the non-thermal effects of microwaves, enhanced effectiveness of anaerobic digestion (AD), and the energy balance of this pretreatment system. Overcoming existing technical challenges, such as the cleavage of extracellular polymers, reducing microwave energy consumption, understanding the non-thermal effects of microwaves, promoting AD of sludge in combination with other chemical and physical methods, and expanding the application of the technology, are the main scientific focuses. Additionally, this paper thoroughly examines both the constraints and potential of microwave pretreatment technology for wastewater treatment.

## Introduction

In treating domestic wastewater using the activated sludge method, approximately 7–10 kg of bioactive sludge, mixing of secondary sludge and waste activated sludge (WAS), is generated as a by-product for every 3 m^3^ of treated wastewater (Bozkurt and Apul 2020). Due to rapid urbanization and population growth, residual sludge production is increasing in many countries. For instance, the annual production of sewage sludge in 15 EU countries increased by almost 50%, from 6.5 million tonnes of dry solids in 1992 to 9.8 million tonnes in 2005 (Kelessidis and Stasinakis 2012). In the US, around 6 million tonnes of dried residual sludge have been generated each year since 2015, with expectations of continued growth (Zhen et al. 2017). China produced 6.25 million tonnes of dry solids in 2013, with projections to reach 39.78 million tonnes by 2020 (Lishan et al. 2018). Anaerobic digestion is a prevalent method for stabilizing sludge, where organic components are transformed into methane through biological processes in an oxygen-deprived environment. However, even with prolonged retention periods (specifically, between 10 and 40 days), a significant portion of organic material (Tyagi and Lo 2011), around 35–45%, exits anaerobic digesters without undergoing digestion (Yuan and Zhu 2016). This issue primarily stems from the properties of WAS, which is a blend containing microbial cells, synthetic and natural organic materials, minerals, and heavy metals, all bound within a polymeric structure of extracellular polymeric substances (EPS) and cations (Gil et al. 2018). The EPS envelops the flocs, while robust cell walls protect the intracellular organic content during AD processing, leading to a deceleration in the hydrolysis stage and resulting in inadequate substrate utilization (Gil et al. 2019).

Before subjecting WAS to anaerobic digestion, hydrolysis can be expedited through various pretreatment approaches such as mechanical (Serrano et al. 2016), chemical (Bougrier et al. 2006; Chu et al. 2009), biological (Barjenbruch and Kopplow 2003; Carvajal et al. 2013), thermal (Eskicioglu et al. 2006; Kuglarz et al. 2013), or a combination of these methods (Toreci et al. 2009; Yu et al. 2010). Among these preparatory techniques, microwave irradiation stands out as an energy-efficient and targeted heating method that can swiftly induce hydrolysis, making it a feasible choice (Tyagi and Lo 2013). Microwave pretreatment, a derivative of traditional thermal pretreatment (Climent et al. 2007) is an emerging technology that has been developed as researchers have studied the biological effects of microwave radiation in depth (Banik et al. 2003). Despite the comprehensive understanding of microwave sludge pretreatment technology in recent years, there are still a number of issues that need to be addressed in its current development.

Numerous studies in the existing literature have documented the positive effects of using microwave pretreatment technology on sewage sludge. Recently, bibliometric analyses (Davarazar et al. 2020) have gained prominence as a method for monitoring and assessing research progress, as well as the contributions of researchers, countries, academic institutions, and global universities in specific domains (Gandia et al. 2019; Hoang et al. 2021). The significance of these crucial and decisive investigations is underscored by the notable increase in the quantity of scientometric research conducted across various scientific disciplines (Olawumi and Chan 2018; Saranya et al. 2018). This paper presents a comprehensive assessment of microwave pretreatment technology for sewage sludge. It conducts a critical review of scientometrics and summarizes all published articles from 1980 to 2023 on the microwave pretreatment of sewage sludge. Additionally, it offers an overview of the technology’s history and current developments in terms of time, region, and research area and highlights both its shortcomings and development prospects in sewage sludge treatment.

## Methodology

This section details the scientometric analysis methods and specific literature screening tools utilized in this thesis, encompassing both scientometric and content analysis. The first part delineates the process of using keywords to search the literature in the Web of Science (WoS) core collection database, including how to filter out less relevant literature records by combining different keywords. The second section elucidates the logic behind the search strategy employed in this paper, critically discussing all the literature obtained in the field using three distinct bibliometric tools: CiteSpace, Scientopy, and WoS analysis.

### Scientometric analysis

Scientometrics, a branch of informatics, involves the quantitative analysis of scientific literature to discern patterns, emerging trends, and the overall knowledge framework within research domains (Azam et al. 2021). Scientific mapping tools typically process scientific publications as input, producing interactive visual representations of complex structures for statistical examination and visual exploration (Yigitcanlar et al. 2020). A variety of scientific mapping tools are available for quantitative analysis, including HistCite (Garfield 2009), VOSviewer (Sgambati and Gargiulo 2022), Scientopy (Ruiz-Rosero et al. 2019), and CiteSpace (Chen et al. 2012). These tools share a common feature: they all generate scientific maps that depict relationships between various elements, offering a spatial representation of how disciplines, fields, participants (authors, institutions, and countries), and individual papers interconnect (Wagner et al. 2011). In this paper, two such scientific mapping software, Scientopy and CiteSpace, are employed to analyze the application of microwave pretreatment to sewage sludge, ensuring a clear and accurate presentation of the results.

The literature on microwave pretreatment of sewage sludge was sourced from the Web of Science (WoS) core collection database using carefully chosen keywords based on a primary literature survey (Table 1). The advanced search function in WoS centered on keywords related to wastewater treatment (Table 1), and the results from each keyword set were combined using the AND operator to pinpoint relevant literature within the field (Table 1). On May 23, 2023, a compilation of English language papers published between 1981 and 2023 was generated based on this search query. The focus was primarily on identifying papers that included the specified keywords in their titles. A meticulous selection process was employed to ensure precision in the analysis, discarding any irrelevant literature. The relevant records were then tagged within the WoS platform and exported in both ‘tab’ and ‘plain text’ formats for further examination using Scientopy and CiteSpace, respectively. This review included an evaluation of the following metrics: publication year, publication type, contributing country, keywords, authors, cited authors, cited journals, and subject area.

**Table 1:** A set of keywords was formulated for conducting a search on the WoS platform, focusing on published documents related to wastewater (sludge) treatment involving microwave pretreatment technology

Database	Set keywords	Results (number of documents)
WoS core collection	#1 T1(Title) = (microwave* or microwave treat* or radiation treatment* or radiation pretreatment*)	151,879
	#2 T2(Title) = (wastewater* or sludge* or waste activated sludge* or wastewater sludge*)	49,774
	#3 TS(Topic) = (sludge pretreatment* or wastewater pretreatment* or microwave pretreatment*)	5737
	#4 #1 AND #2 AND #3	1763

The table includes an asterisk to indicate the inclusion of additional potential keywords, aiming to expand the search scope and reduce the likelihood of missing any pertinent keywords denoted by various letters.

### Content analysis

**Fig. 1:**
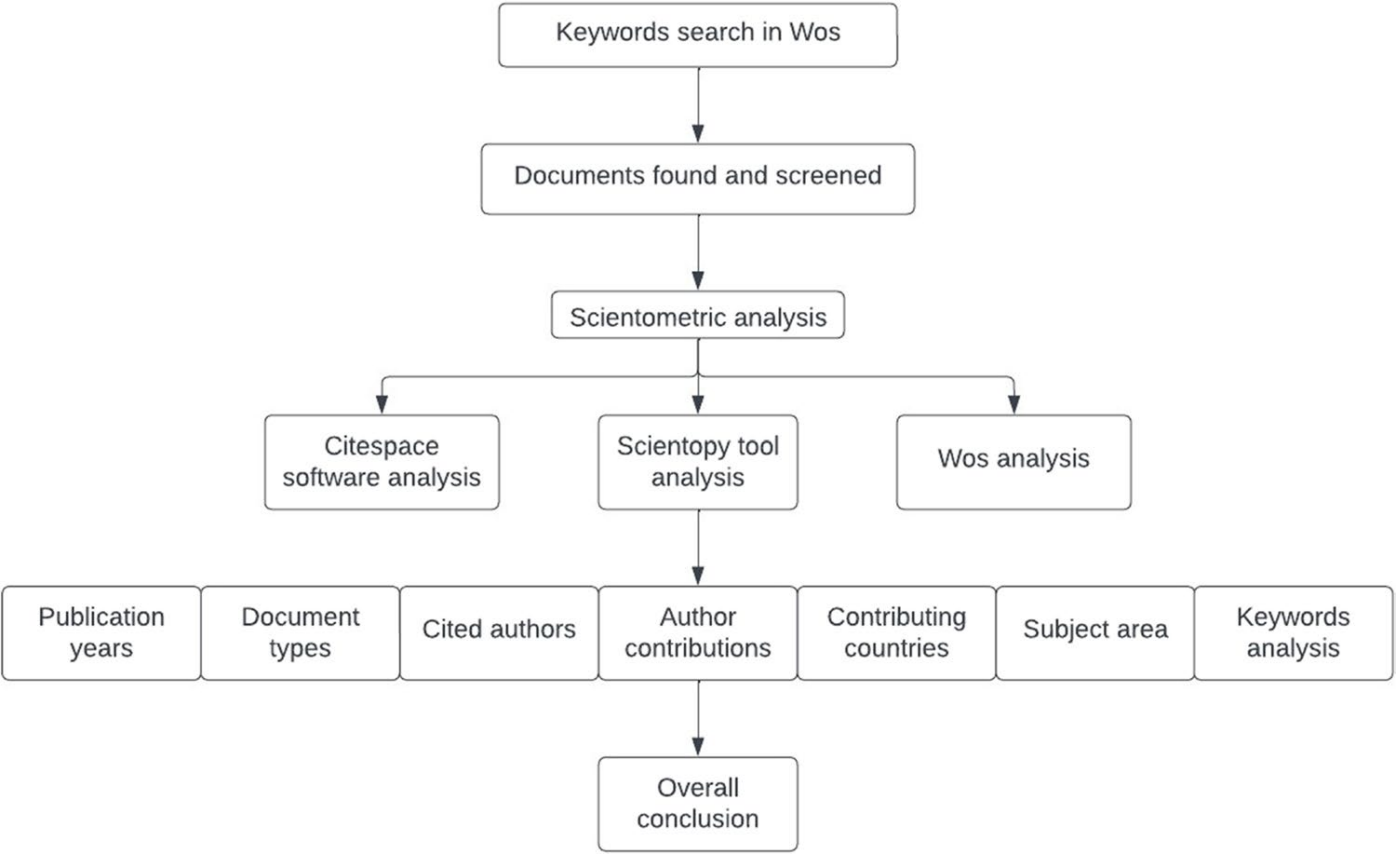
A schematic of the research design

A comprehensive exploration of the published papers necessitates an in-depth scientometric examination. This endeavour aims to unveil past and present research trends concerning microwave pretreatment in sewage sludge treatment. The scientometric tools ‘Web of Science,’ ‘Scientopy,’ and ‘CiteSpace’ serve as foundational resources to facilitate insightful discourse on prevailing and forthcoming research focal points, as well as existing gaps that await resolution through future investigations. The research design of this paper is visually depicted in Fig. 1. Utilizing three distinct bibliometric analyses, this study will scrutinize the entire assemblage of literature records in this domain, dissecting aspects such as publication year, authors, countries, research domains, and keywords. Based on these findings, the paper will identify prevailing deficiencies and chart potential pathways for the evolution of microwave pretreatment technology in sewage sludge treatment.

## Results and discussion

The results obtained are discussed and analyzed in this section in three parts. The analysis of the documents includes a categorization of the obtained documentary records in terms of chronological order and document type. Changes in research focus and format over time are summarized, and a conclusion of the highly cited literature in the field is presented to reveal hotspots in microwave pretreatment of sewage sludge. Regarding contributions, the paper discusses the input made to the field by authors, national and research institutions, and journals, respectively. The results of various levels of contribution to the research in this field are visualized using various scientometric tools. Finally, by analyzing trends in different research areas and keywords over time, two major challenges are identified: the mechanism of microwave non-thermal effects and the high energy consumption.

### Analysis of documents

**Fig. 2:**
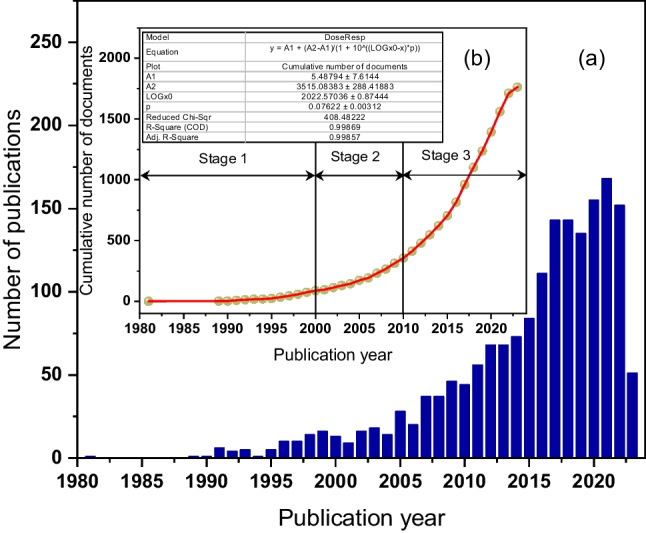
**a** The count of papers pertaining to the application of microwave pretreatment technology for wastewater sludge treatment per annum within the span of 1980 to 2023. **b** The accumulated tally of publications encompassing the interval from 1980 to 2023

Figure 2a illustrates the annual number of publications on the application of microwave pretreatment of sewage sludge. The publications in this field arranged chronologically can be divided into three stages. The first stage marks the inception of microwave pretreatment technology (Stage 1), with the earliest publication dating back to 1981 by Atsuya and Akatsuka (1981). In this article, microwave technology was employed to enhance the accuracy of trace element determination in sludge, using microwave pretreatment to expedite the digestion time of sewage sludge and achieving 92–101% recovery for arsenic determination. Colombini et al. (1998) conducted a study where sludge samples underwent digestion in a microwave oven using an alkaline persulfate solution, followed by analysis for total phosphorus and nitrogen via ion chromatography without preliminary sample treatment. The results demonstrated notable reproducibility and accuracy within the standard concentration range used for wastewater assessment. While not specifically using microwave technology as a pretreatment to enhance anaerobic digestion results, the application of microwave treatment to improve sludge solubility and reduce digestion time has been widely utilized (Pérez-Cid et al. 1999; Santos et al. 2000).

Since 2000 (Stage 2), researchers have increasingly focused on microwave technology as a pretreatment prior to anaerobic digestion (AD), comparing it with other sludge pretreatment technologies. Hong et al. (2004) investigated the effect of microwave radiation versus external heating on pathogen destruction in sewage sludge. They found that cell membranes are damaged with increasing intensity and temperature of microwave radiation, and at the same temperature, microwave radiation more effectively reduces cell activity than external heating, almost ceasing bacterial activity at temperatures above 68 °C. A similar study by Pino-Jelcic et al. (2006) concluded that microwave/digested sludge showed fewer faecal coliforms and Salmonella spp. compared to conventional addition, noting that microwave heating enhanced biodegradability under thermophilic AD and improved the dewatering rate of digested sludge. Eskicioglu et al. (2007b) investigated various sludge concentrations under microwave irradiation at low temperatures (50–96 °C), focusing on the dissolution of activated sludge and cumulative biogas generation through anaerobic digestion. Their findings indicated a significant increase in soluble total chemical oxygen demand (SCOD/TCOD) for high and low sludge concentrations, alongside improved dewatering efficiency of microwave-treated sludge after anaerobic digestion. Zheng et al. (2009) demonstrated that microwave pretreatment at 90 °C for primary sludge with a total solids concentration of 4% led to a 37% increase in the biogas production rate compared to non-pretreated sludge, with their model indicating an increase in biogas yield factor as microwave pretreatment temperature increased.

After 2010 (Stage 3), the publication of papers on microwave pretreatment, particularly for AD and sludge dewatering, increased rapidly. Researchers focused on enhancing microwave energy efficiency and the factors influencing microwave pretreatment. Jackowiak et al. (2011) aimed to optimize microwave pretreatment of wheat straw, with findings indicating a 28% increase in methane yield at 150 °C compared to untreated samples. They also highlighted the need for a positive energy balance, suggesting that microwave equipment power consumption should not exceed 2.65 kJ/g tVS. Uma Rani et al. (2013) found that microwave irradiation reduced the initial lag time of AD, with the best energy-efficient pretreatment lasting 12 min at 70% intensity. Studies also explored microwave pretreatment’s role in different substrates. For instance, Passos et al. (2014) reported a 30% increase in methane production from microalgal biomass, and Tyagi et al. (2014) documented a significant enhancement in sludge from pulp and paper mills following alkali-enhanced microwave pretreatment. Srinivasan et al. (2016) demonstrated high treatment efficiency and low energy requirements in a dairy manure treatment system using microwave and hydrogen peroxide pretreatment. In terms of main influencing factors, Tas et al. (2018) noted microwave pretreatment’s advantages over ultrasonic in methane yield improvement, highlighting economic considerations and the need for large-scale experiment data. Alizadeh et al. (2018) used a mathematical model to fit methane yield and solubilization efficiency, showing that microwave pretreatment enhances AD of kitchen waste by destroying its recalcitrant structure, thereby increasing biogas production, with irradiation time and temperature as key factors.

It is noteworthy that the accumulation of publications within this scientific domain follows a Sigmoidal growth trajectory, as clearly depicted in Fig. 2b, with a high coefficient of determination (*R*2 = 0.9987). This pattern suggests that the field has yet to reach a definitive stage of maturity, indicating that ongoing research is still actively seeking to fill the existing gaps in knowledge regarding microwave pretreatment for wastewater treatment.

**Fig. 3:**
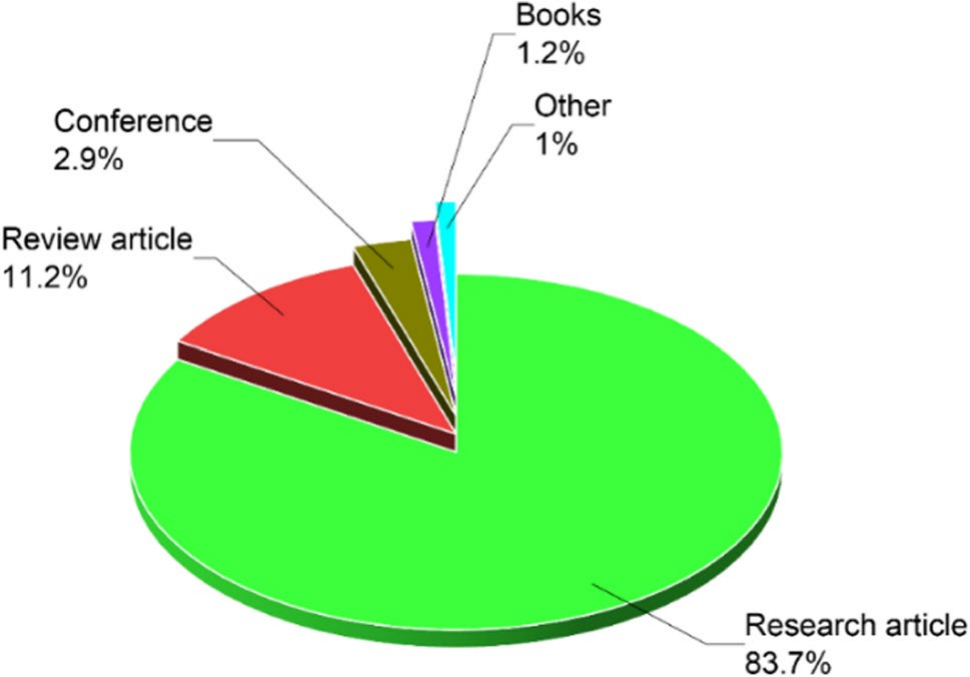
Types of published papers on the use of microwave pretreatment technology for sewage sludge treatment

Figure 3 provides a detailed overview of the categories of publications related to the use of microwaves in water and wastewater treatment. It is apparent that research articles are the most common type, accounting for 83.7% of publications, followed by reviews (11.2%), conference abstracts (2.9%), book chapters (1.2%), and other relevant documents such as patents and technical notes. Table 2 summarizes the key conclusions and findings derived from the analyzed papers.

**Table 2:** The paramount discoveries from published papers concerning the viability of microwave pretreatment in sewage sludge treatment, arranged based on their citation counts from the Web of Science (WoS)

Scope of the study	Main findings/remarks	Ref
Comparison of the effects of different pretreatment effects on enhanced sewage sludge decomposition and subsequent AD	In addition to improving methane recovery from subsequent AD of sludge, microwave pretreatment is also effective in destroying pathogens and the ability to dewater sludge. However, there is still a lack of research on the mechanism of microwaves, and the non-thermal effects of microwaves are highly controversial	(Zhen et al. 2017)
The effect of non-thermal effects of microwave on improving the digestibility of waste activated sludge	The non-thermal effect of microwaves is not reflected in the solubility of the waste activated sludge, but the final methane yield reflects the non-thermal effect of microwaves	(Eskicioglu et al. 2007a)
Microwave irradiation for resource recovery and sludge treatment	Microwave heating is more effective than conventional heating in that it reduces reaction time, enhances sludge solubility and subsequent AD, and improves sludge dewatering and has a significant inactivating effect on pathogens in the sludge. Also, microwave radiation can improve the recovery of nutrients from the sludge and has a great advantage in producing a clean environment	(Tyagi and Lo 2013)
Improving sludge methane potential for pulp mill wastewater treatment using microwave pretreatment technology	Compared to ultrasonic pretreatment, microwave pretreatment is more advantageous in increasing the biodegradation rate. However, for mixed sludge, microwave pretreatment is not energy-economical due to the high energy input required	(Saha et al. 2011)
Microwave heating pretreatment for bioenergy applications	Microwave heating technique can be a sustainable and energy-efficient option for effective biomass conversion to biofuels and biochemical conversion. However, the main factors limiting the development of this technology are the cost of processing and the technology base	(Kostas et al. 2017)
Effect of microwave pretreatment on the co-digestion of food waste and sewage sludge	The methane production of sewage sludge (SS) was higher than that of food sludge (FW) after microwave pretreatment of the two types of sludge individually. However, anaerobic co-digestion based on microwave pretreatment was more effective for methane production after mixing the two sludges at 3:2 (FW: SS)	(Zhang et al. 2016)
Effect of mixed microwave irradiation and acidification treatment on the dewatering performance of wastewater	The microwave heating temperature and pH value play a key role in improving the dewatering performance of sludge, with higher temperatures facilitating the disintegration of sludge flocs. The combination of microwave and acid treatment significantly reduces the bound water content and facilitates deep sludge dewatering	(Liu et al. 2016)
Effect of microwave irradiation and low temperature thermal pretreatment on sludge dissolution and subsequent AD at the same temperature range	The application of microwave radiation prior to AD is superior to thermal pretreatment, as confirmed by the release of sludge components and the increase in methane production. Also, microwave pretreatment prior to AD ensures the absence of Salmonella spp. and a 50% reduction in *E. coli* as well as *Clostridium perfringens*	(Kuglarz et al. 2013)
Effect of microwave radiation pretreatment of municipal secondary sludge on AD	Microwave pretreatment technology aids the breakdown of sludge particles, which leads to an increase in the amount of intracellular material in the medium and improves the AD of the sludge. Also, microwave pretreatment reduces the hydraulic residence time of AD and increases the final biogas production	(Park et al. 2004)
Combined microwave-acid pretreatment disintegration of macroalgae *L. japonica* biomass with dark fermentation for hydrogen production in batch mode	Microwave acid treatment breaks down large algal cells and releases intracellular organic matter, and hydrogen production after microwave pretreatment is increased by a twofold increase compared to a blank control test	(Yin and Wang 2018)

Table 2 summarizes key findings from numerous studies on microwave pretreatment in sewage sludge treatment, encompassing aspects such as effects on sludge decomposition, pathogen destruction, methane production, dewatering performance, and nutrient recovery. The table reveals that microwave pretreatment can enhance methane recovery, improve dewatering, and inactivate pathogens, although the non-thermal effect of microwave and the mechanism remain a subject of controversy. Comparative analysis of microwave pretreatment’s efficiency with methods like ultrasonic pretreatment underscores significant energy and cost considerations.

Given these findings, it is apparent that microwave pretreatment offers considerable potential for sewage sludge treatment, outperforming traditional methods in several respects. Nevertheless, the associated high energy requirements and operational costs pose substantial challenges. The ongoing debate concerning the non-thermal effects and mechanisms of microwaves highlights an urgent need for further research, especially in the context of scaling these effects for industrial-scale applications. Future studies should aim to integrate microwave pretreatment with other emerging technologies to boost its efficiency and feasibility. Additionally, a thorough exploration of the economic and environmental implications of adopting this technology on a larger scale is essential for its practical application and to ensure alignment with sustainability objectives.

### Scientometric analysis of authors, countries, and journal contributions

In this section, a comprehensive analysis of various publications related to the use of microwave pretreatment in sewage sludge treatment is presented. This analysis includes an examination of the authors, countries of origin, affiliated organisations, and sources of the publications. Analyzing these elements can reveal insights into evolving trends and the formation of international partnerships in the development of microwave pretreatment research technology globally (Guiling et al. 2022). Furthermore, it provides data to support further quantitative analysis (Rosokhata et al. 2021) for a general overview of the field’s development.

#### Contribution of authors

**Fig. 4:**
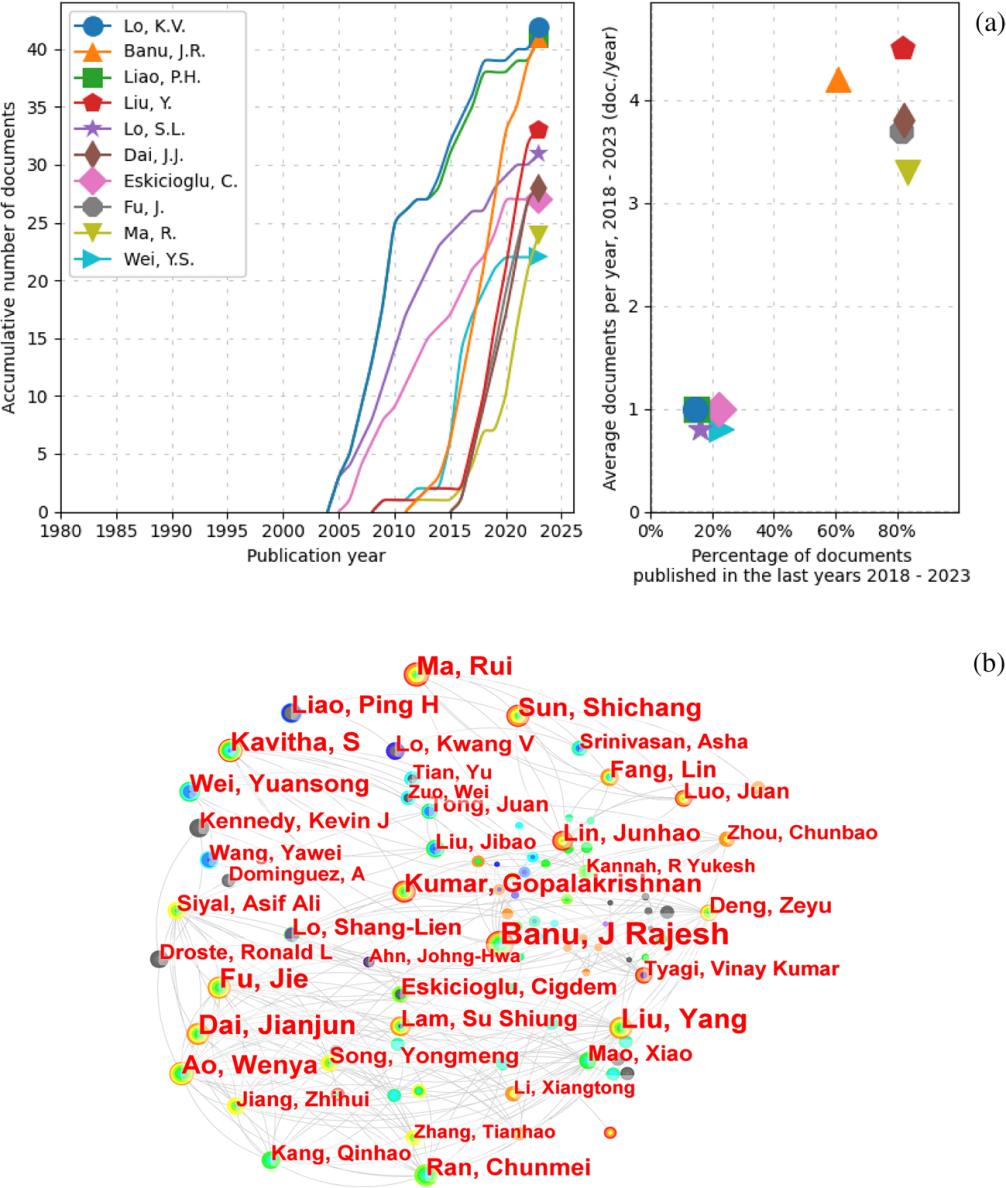
**a** The roles and contributions of authors in scientific publications concerning microwave pretreatment of wastewater sludge, as gathered through Scientopy. **b** CiteSpace outcomes reveal collaborative efforts among diverse authors in this field. The font size corresponds to the extent of an author’s contribution, with larger fonts indicating greater involvement

Figure 4 illustrates the frequency of publications on microwave pretreatment of sewage sludge. The CiteSpace and Scientopy results (shown in Fig. 4a, b, respectively) identify major contributors in the field such as Lo, K.V. (Lo et al. 2015) and Liao, P.H. (Lo et al. 2018) from Canada, with 42 papers; Banu, JR (Ebenezer et al. 2015) from India, with 41 papers; Liu, Y. (Yang et al. 2013) with 33 papers; and Lo, S.L (Tyagi and Lo 2013) from China, with 31 papers. Figure 4a (left) highlights the papers published in the last five years, indicating these significant contributors have remained active (Ruiz-Rosero et al. 2019). Additionally, there are several authors who have published multiple articles recently. For instance, Dai et al. (Zhang et al. 2017) studied microwave pyrolysis of textile printing and dyeing sludge, Fu et al. (Ao et al. 2018) reviewed the efficiency of activated carbon preparation under microwave radiation, Ma et al. (Ma et al. 2017) explored the impact of catalysts at varying temperatures on the conversion of organic matter and biofuel production by microwave pyrolysis of sludge, and Wei et al. (Niu et al. 2019) investigated microwave pretreatment combined with zero-valent iron technology to enhance AD. This demonstrates the popularity and growth of microwave pretreatment of sludge as a research area.

**Fig. 5:**
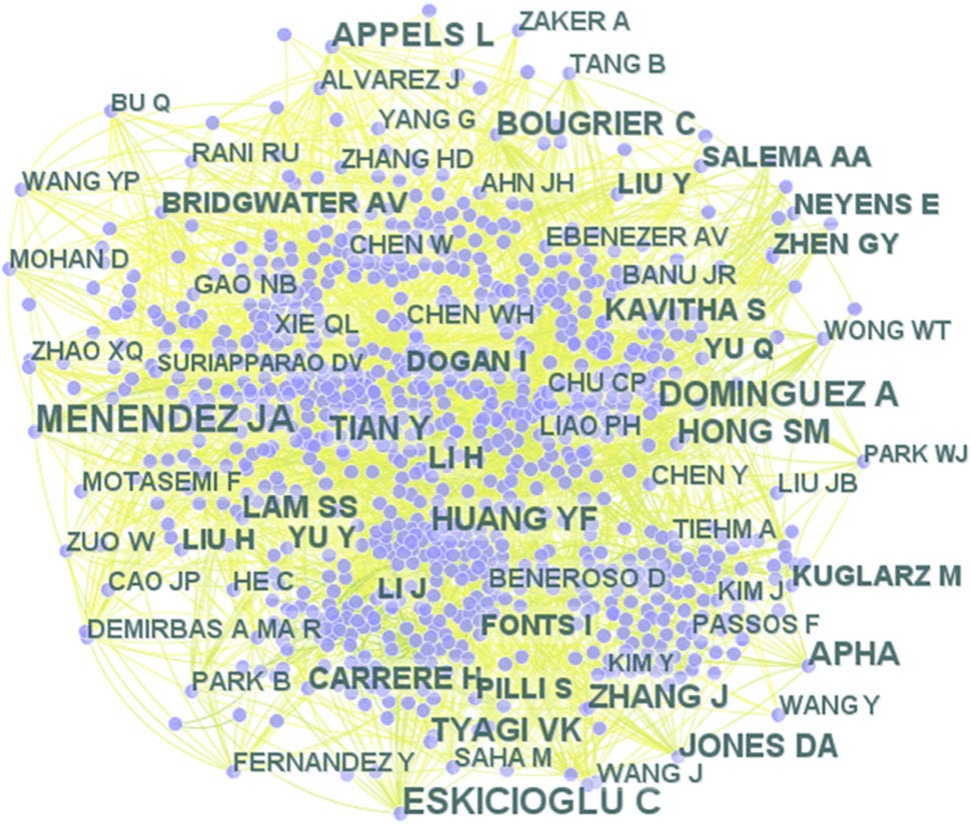
Author’s contributions to the number of citations for publications on microwave pretreatment of sewage sludge

Apart from the volume of publications, the number of citations an author receives is another metric reflecting their influence on advancing scientific knowledge within the field (Zhang et al. 2022). As depicted in Fig. 5, Cigdem Eskicioglu and collaborators (Kor-Bicakci et al. 2020) are the most cited researchers in this field. While extensive research on microwave pretreatment has been conducted, its application remains predominantly at the laboratory scale. Greater adoption will necessitate further pilot investigations to address existing challenges. For example, Appels et al. (2013) examined the effects of microwave pretreatment using a pilot-scale semi-continuous digester set-up, finding a 50% higher average biogas yield compared to a blank test, though they also highlighted considerations of energy efficiency. Thompson et al. (2019) demonstrated that microwave pretreatment improved the efficiency of saccharification and fermentation using brown algae but noted the need for substantial capital investment and energy input, with plans for future pilot-scale studies. Atelge et al. (2020) also emphasized that while microwave radiation is still in the developmental stage and applied at batch or pilot scale, transitioning to full scale requires transforming the technology from research to a mature technology.

The comprehensive analysis of contributions in the field of microwave pretreatment of sewage sludge underscores a significant trend towards innovative and efficient waste management techniques. While the prolific output of key researchers such as Lo K.V., Banu JR, and Liu Y. demonstrates a robust academic interest, the prevailing focus on laboratory-scale studies signals a gap in the translation of this research into practical, large-scale applications. The high citation rates of works by Cigdem Eskicioglu and others reflect the academic community’s recognition of their valuable insights, yet the field appears to be at a critical juncture. The transition from laboratory to pilot-scale studies, as explored by Appels et al. and Thompson et al., is a crucial step that demands not only scientific rigor but also considerations of economic feasibility and energy efficiency. The future of microwave pretreatment in sewage sludge management hinges on bridging these gaps, transforming research into mature technology capable of addressing real-world environmental challenges. This necessitates a multidisciplinary approach, combining scientific innovation with practical engineering solutions, policy support, and sustainable economic models to realize the full potential of this technology in contributing to environmental sustainability.

#### Contribution of countries and research organizations

**Fig. 6:**
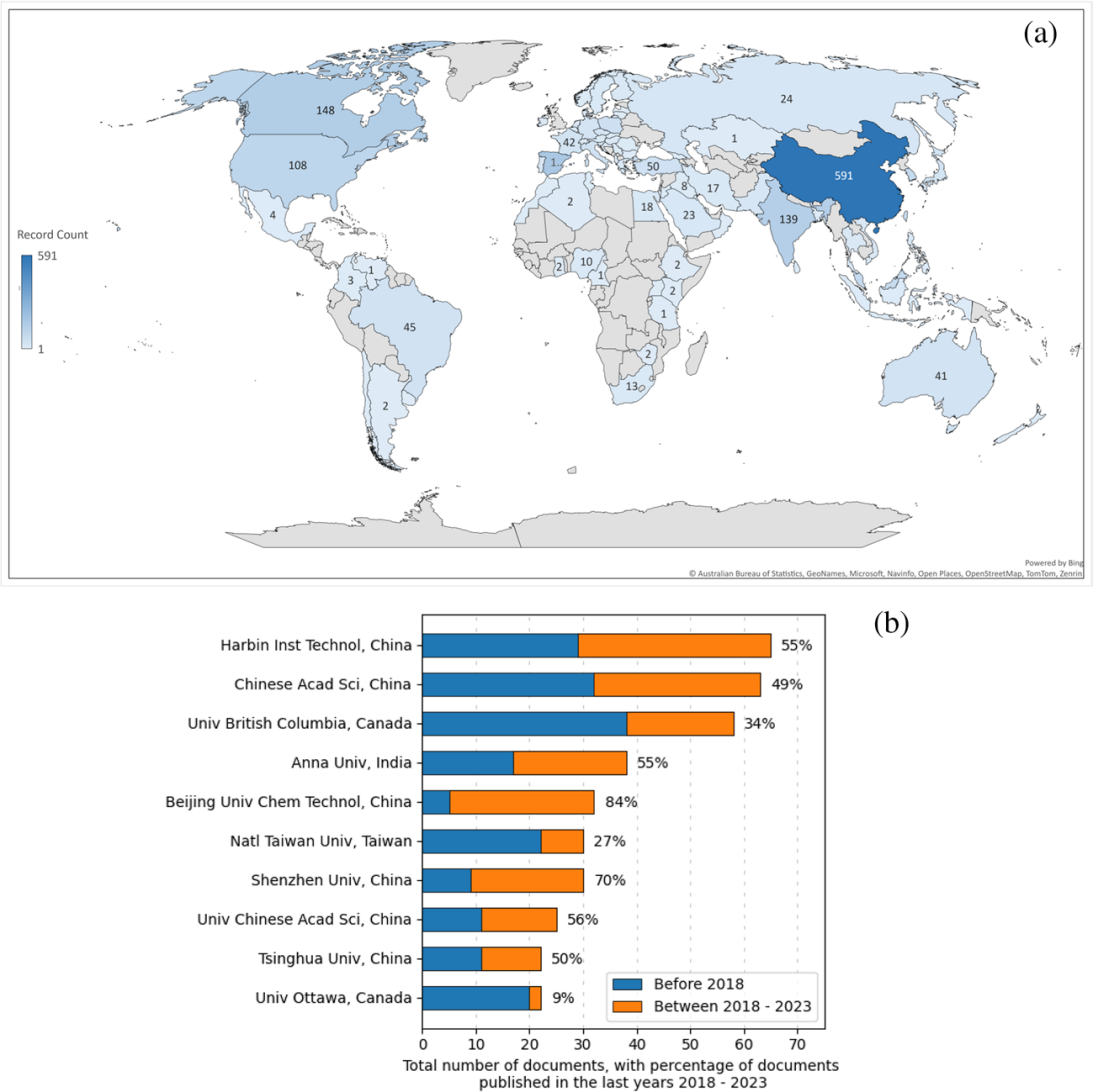
**a** The involvement of countries and research organizations in the implementation of microwave pretreatment for sewage sludge is depicted. **b** Examination utilizing Scientopy provides further insights. Contribution of journals

Figure 6 illustrates the delineation of contributions from various countries and organizations to the domain of microwave pretreatment in sewage sludge treatment. According to Fig. 6a, China (591 records), Spain (175 records), and Canada (148 records) have published the highest number of papers in this field. In addition, the scientific output of leading national organizations, as shown in Fig. 6b, has increased significantly since 2018, and new national organisations have started to gradually join the research in this field in recent years. For example, the Harbin Institute of Technology (China) and the Chinese Academy of Sciences (China) have published 55% and 49% of the scientific literature related to microwave pretreatment of sludge in the last five years, respectively. Anna University (India) has also made a significant contribution in the field of microwave pretreatment of sewage sludge in the last 5 years, with 55% of the published literature. Furthermore, according to the information provided in Fig. 6, Chinese and Indian research and development organizations are currently the main players in this research area. Also, through publications in recent years, it can be understood that the main research topics of these organizations are currently focused on the use of different catalysts to synergistically increase the heating rate of microwaves (Li et al. 2022b; Xie et al. 2022; Lu et al. 2023), the use of microwave-assisted technology to enhance the energy conversion rate of bio-waste (Yang et al. 2022; Chandrasekaran and Chithra 2022; Usmani et al. 2023), and microwave pretreatment technology to enhance hydrogen production from sludge (Dinesh Kumar et al. 2020; Zhao et al. 2023). This bodes well for the future development of sludge pretreatment in favor of industrialization and energy efficiency.

**Fig. 7:**
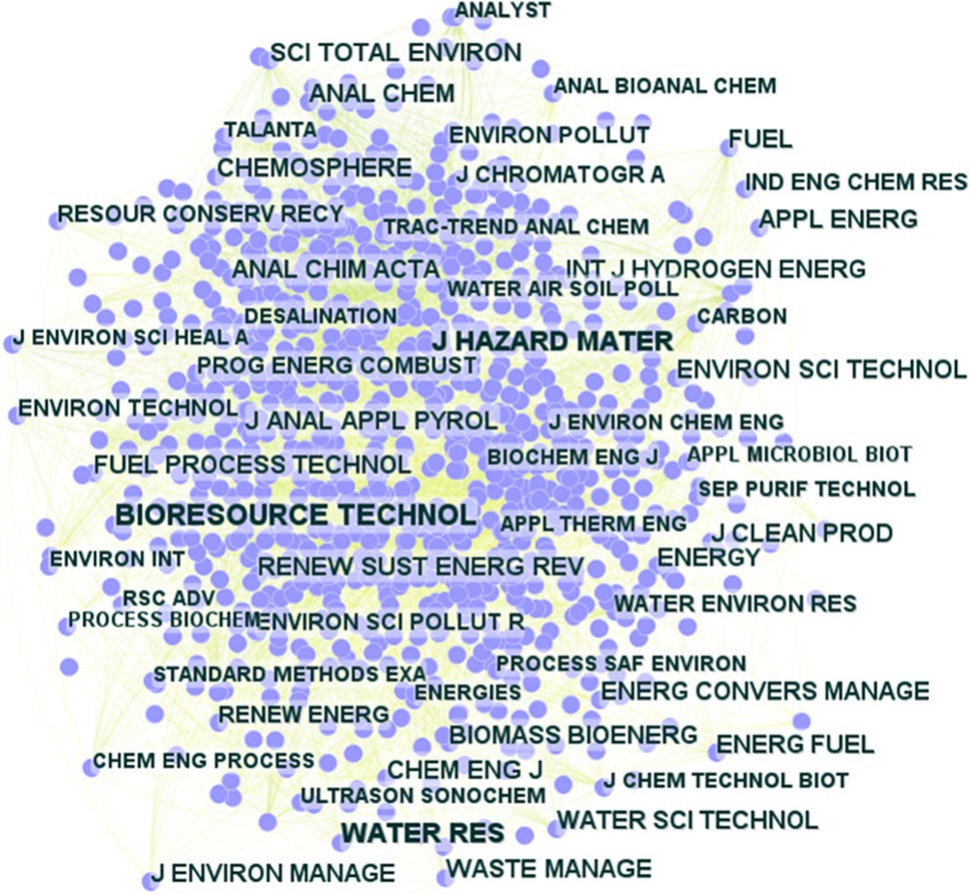
Contribution of various journals to the number of citations of publications on the use of microwave pretreatment of sewage sludge

In terms of publication sources, an analysis via WoS indicates that ‘Bioresource Technology,’ ‘Water Research,’ and ‘Journal of Hazardous Materials’ are the leading journals in this area, with 248, 241, and 242 papers, respectively. Figure 7 visually represents the contributions of scientific journals to the research landscape in microwave pretreatment technology. However, it is important to note that the correlation between the number of published papers and citations received is not always direct. In some cases, journals may publish fewer papers but receive a considerable number of citations. For example, ‘Environmental Science and Technology’ has published 131 papers on microwave pretreatment, yet it holds the highest average citations per article (101.79) among all journals in this field.

The international landscape of microwave pretreatment research, dominated by Chinese and Indian organizations, underscores the global recognition of the importance of sustainable waste management. The significant involvement of countries and organizations in advancing this technology highlights a collaborative effort towards environmental sustainability. The focus on innovative research topics like catalyst use for increased heating rates, energy conversion enhancement, and hydrogen production from sludge reflects a progressive approach towards addressing energy and environmental challenges. This trend towards specialisation in microwave pretreatment indicates a promising future for this technology, potentially leading to industrial-scale applications. However, the successful transition from laboratory research to industrial application will require a concerted effort in overcoming technical, economic, and regulatory challenges. This necessitates not only continuous scientific innovation but also the need for international collaboration, policy development, and investment in pilot projects to facilitate the practical implementation of these research findings. The integration of these diverse elements will be crucial in realizing the full potential of microwave pretreatment technology in contributing to a sustainable future.

### Trends in microwave pretreatment technology

**Fig. 8:**
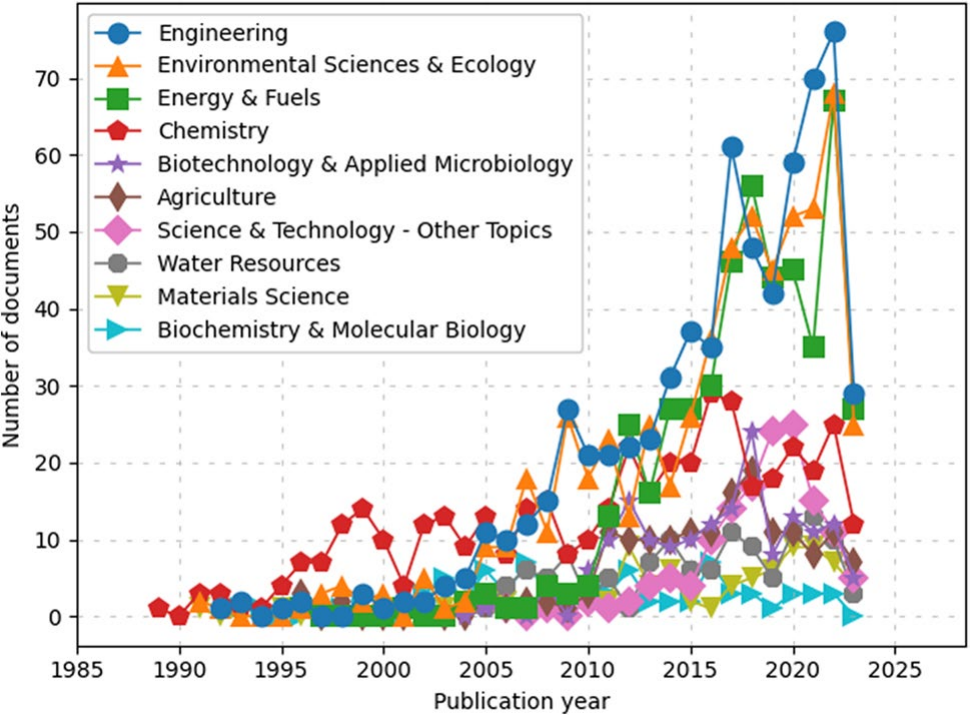
Subject area for wastewater treatment using microwave pretreatment

Keyword and subject area analysis reveals that microwave pretreatment is a major trend in sewage sludge applications (Macías-Quiroga et al. 2021). Figure 8 illustrates the diverse nature of research in this domain, covering a range of disciplines, including engineering, environmental science, energy, chemistry, biotechnology, agriculture, water resources, and, to a lesser extent, materials science and biochemistry.

**Fig. 9:**
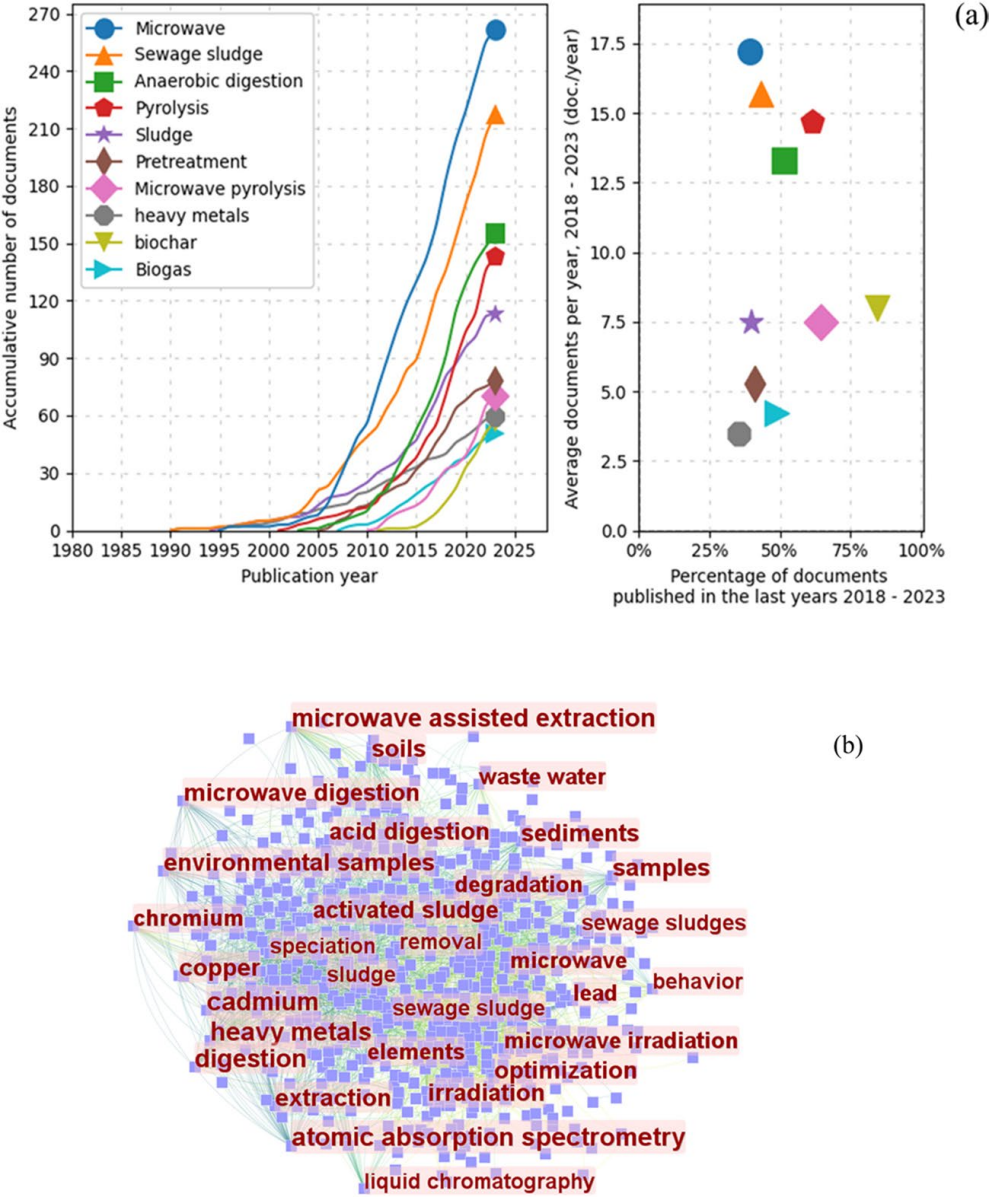
The most important keywords found in published documents on microwave pretreatment of sewage. **a** Analysis results from Scientopy. **b** Analysis results from Citespace

Figure 9a identifies key keywords in papers on microwave pretreatment of sewage sludge, highlighting hot topics such as enhancing anaerobic digestion (AD), microwave pyrolysis mechanisms, combined catalyst use, and biogas production. These topics are consistent with previous findings on national and research institute contributions, indicating a coherent research trend (refer to “Contribution of countries and research organizations”). The keyword search results using CiteSpace (Fig. 9b) offer an intuitive insight into current hotspots in microwave pretreatment research (López-Serrano et al. 2020). In addition to the previously mentioned topics, new keywords like optimization, degradation, and behavior have emerged. These are related to research on improving biogas purity through microwave pretreatment (Wang et al. 2022; Luo et al. 2023), enhancing sludge hydrolysis post-microwaving (Cheng et al. 2023), and the destruction of cellulose in sludge (Kazawadi et al. 2022).

The timeline visualization is designed to illustrate the evolution of key trends in the research domain. It includes aspects such as the emergence of keywords within clusters, the timing of their introduction, the changing significance of clusters over time, and the identification of symbolic keywords, particularly those with high and medium centrality (Zhang et al. 2023). This visualization aids in comprehending the developmental trajectory and current focus areas within the field.

**Fig. 10:**
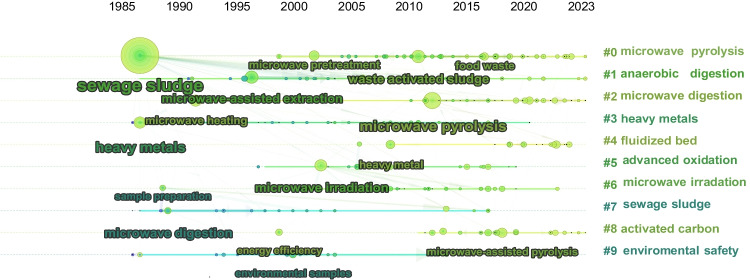
Trends in the occurrence of keywords obtained using CiteSpace for microwave pretreatment of sewage sludge

In the context of keyword analysis focused on microwave-pre-treated sludge, Fig. 10 illustrates the development of the initial 10 clusters. Among these, clusters such as (#0) microwave pyrolysis, (#1) anaerobic digestion, and (#2) microwave digestion have consistently garnered significant attention since their introduction in this field. Emerging themes like microwave co-pretreatment, sludge drying, and the immobilization of heavy metals in sludge have evolved from earlier clusters. Notably, from 1981 until approximately 2014, sewage sludge was the primary focus of microwave pretreatment research, with a gradual shift towards exploring reaction mechanisms and optimising effects in more recent years. Table 3 summarizes the latest advancements in microwave pretreatment for sludge.

**Table 3:** Recent findings and prospects

Pretreatment system	Pollutant type	Efficiency	Remarks	Ref
Microwave combined with thermal-alkaline	Municipal sewage sludge	The extraction efficiency of sugars from sewage sludge was evaluated under different microwave pretreatment combined with thermal-alkaline conditions. Crude sugar yields and extraction efficiencies increased with increasing amounts of NaOH	Microwave pretreatment can improve the extraction rate of crude sugar from sludge, but when the microwave intensity is increased, the microwave time is extended or the amount of NaOH is increased, the purity of crude sugar will then decrease	(Cheng et al. 2023)
Microwave	Fruit and vegetable waste (FVW) mixed with anaerobic sludge (AS)	Microwave pretreatment increases the daily and cumulative biogas yield as well as the methane yield from FVW and AS co-digestion. This also improved the stability of the digester by increasing the buffering capacity of the digester, AS: FVW (25:75) had the highest nutrient dissolution and 10% methane yield with microwave pretreatment at 300 W	Co-digestion and microwave pretreatment make hydrolysis no longer the limiting step in AD, but methane production the new limiting step, and how to improve the final methane yield and reduce energy efficiency costs becomes a new challenge	(Agrawal et al. 2023)
CaO_2_-assisted microwave pretreatment	Maize straw	CaO_2_-assisted microwave pretreatment effectively reduced the crystallinity of maize straw and increased the initial water-soluble carbon (WSC) content and lignocellulose degradation. Degradation of lignin, cellulose, and hemicellulose reached 25.48%, 43.38%, and 66.29%, respectively	The addition of CaO_2_, although it increases the degradation of lignin by microwave pretreatment, creates residues in the maize straw sludge that can be a burden on subsequent residual sludge treatment	(Lu et al. 2023)
Combined microwave and hydrogen peroxide pretreatment	Tannery sludge	Due to the high content of macromolecular organic matter in tannery sludge, microwave pretreatment can be used to break it down and recover not only bio-based chemicals (e.g., short-chain fatty acids), but also biofuels from the sludge	Although microwave pretreatment can recover most of the polluting waste from tannery sludge, this treatment method is only at the laboratory stage and the industrialization and continuity of the treatment system still requires a lot of research	(Tuci et al. 2022)
Microwave and CaO co-pyrolysis	Sewage sludge and water hyacinth	Microwave pretreatment facilitates the reduction of activation energy and promotes pyrolysis reactions. The addition of CaO reduced the yields of acids and sugars by 10.52% and 5.08%, respectively. Meanwhile, aldehydes and ketones became the main products, accounting for 20.05% and 21.12%, respectively	Extending the microwave pretreatment time can reduce the activation performance of the sludge even more, but at the same time the energy efficiency costs will increase. A balance needs to be found between energy efficiency costs and product yield	(Wei et al. 2022)
Microwave radiation and biomass ash pretreatment	Municipal sewage sludge	The mechanism of microwave radiation and biomass ash conditioning of sludge is different. Microwave radiation destroys the original stable structure of the sludge, allowing more water to flow out. The addition of biomass ash then rebuilds the porous skeletal structure and makes it easier to remove water. Microwave radiation combined with biomass ash pretreatment makes up for the lack of conditioning alone and is significantly better than its pretreatment alone	Combined pretreatment technologies can increase sludge dewatering capacity. However, the addition of biomass ash to microwave pretreatment increases costs and reduces efficiency when the biomass ash exceeds 10% of the total	(Gahlot et al. 2022)
Microwave	Elephant grasses	Increases in microwave process parameters, such as residence time and microwave radiation energy, increase the degradability and bioavailability of fermentable sugars to a reasonable point before they decline	Microwave intensity is more likely to affect the degree of lignin and cellulose cleavage in sludge than changes in microwave duration. The kinetic model can be used to predict the degree of disintegration of lignocellulosic material after microwave pretreatment at any residence time and microwave energy and can be extended for industrial large-scale production of biogas	(Ude and Oluka 2022)

The keyword and subject area analysis of microwave pretreatment research reveals a multidisciplinary convergence, highlighting the field’s complexity and its far-reaching implications. The evolution from a focus on sewage sludge to exploring various reaction mechanisms and optimization techniques indicates a maturing research area. As new keywords like ‘optimisation,’ ‘degradation,’ and ‘behavior’ emerge, they reflect the shifting focus towards enhancing operational efficiencies and understanding deeper scientific processes. However, as the field advances, it faces the challenge of integrating these diverse scientific insights into practical, scalable solutions. This integration requires a balance between innovative research and the pragmatic challenges of implementation, including economic viability and environmental impact. The future direction of this research, therefore, hinges on a synergistic approach that combines scientific discovery with real-world applicability, ensuring that the benefits of microwave pretreatment extend beyond theoretical research to tangible environmental and societal impacts.

#### Microwave non-thermal effects

Microwaves can interact with flocculants in sludge, releasing bound organic matter into solution and breaking down extracellular polymers (Kuglarz et al. 2013). They shield the cell wall within the microflocculant assembly and release intracellular organic matter through three pathways: thermal (Tang et al. 2010), non-thermal (ESKICIOGLU et al. 2007b), and catalytic oxidation (Quan et al. 2007). Thermal effects include solubilization of organic matter, such as denaturing membrane proteins and releasing intracellular organelles, and exceeding the boiling point of intracellular fluids, potentially leading to cell wall rupture (Atkinson et al. 2019). High temperatures can reduce the solubility of gases, forming gas domains (bubbles) in the slurry that may exert additional pressure on cell walls upon bursting. Conversely, high temperatures (70–180 °C) can lead to the polymerisation of low molecular weight sugars and amino acids through the Maillard reaction, resulting in the formation of recalcitrant polymeric organic compounds, potentially reducing the anaerobic digestibility of the resultant product (Eskicioglu et al. 2007a; Toreci et al. 2011).

Non-thermal effects, such as specific effects of electromagnetic radiation, are caused by the rapid oscillation of polar and polarizable molecules or polarized side chains of macromolecules attempting to align with incident electromagnetic waves (Yeneneh et al. 2015). Microwave energy converts to heat through internal rotational resistance, potentially leading to bond breakdown and reorientation. Despite postulations in the literature about non-thermal microwave decomposition pathways, conclusive evidence remains scarce. Experimentally isolating non-thermal effects is challenging, as conventional heating principles differ from microwave radiation, and internal molecular-level temperature monitoring is not straightforward (Kostas et al. 2017). Rao et al. (2022) explored microwave non-thermal effects by examining sludge cake pore structures and analyzing fractal dimensions, suggesting improvements in sludge dewatering were due to non-thermal effects on drainage pore structure and moisture distribution. Conversely, Park and Ahn (2011) and Dai et al. (2013) found no substantial non-thermal effects when comparing microwave pyrolysis with conventional heating in terms of biogas yield, SCOD/TCOD ratios, and emissions of PCDD/Fs. Sólyom et al. (2011) also observed similar biogas production results with both heating methods, indicating a lack of non-thermal microwave effects. Therefore, using microwave pretreatment alongside conventional heating methods to validate the existence of non-thermal effects might not be reliable. More conclusive evidence could potentially be obtained through microstructural observations of sludge or a combination of simulation and experimentation.

#### Energy consumption

Microwave pretreatment of sewage sludge, especially at the laboratory scale, demonstrates significant potential for enhancing biogas production when integrated with anaerobic digesters. This enhancement is attributed to both the thermal (Ahn et al. 2009) and non-thermal (Tyagi and Lo 2013) effects of microwave processing that disrupt the complex floc structure of the sludge. This disruption unfolds and denatures complex organic molecules, including intracellular and extracellular components, making them smaller and more biodegradable (Li et al. 2022a). Such a process results in a notable increase in SCOD, a critical factor in boosting biogas production during subsequent anaerobic digestion. Research (Yu et al. 2010) has shown that microwave irradiation (total irradiation energy of 630 kJ) can raise the SCOD/TCOD ratio in sludge from 2 to 22%. Similarly, in WAS, this ratio increased from 8 to 18% after microwave heating at 72.5 °C and from 6 to 18% following treatment at 96 °C. Gil et al. (2019) reported an increase in solubility (COD/TVS ratio) of floating sewage sludge ranging from 43 to 66%, depending on the total energy applied and the power rating.

In addition to the increase in SCOD content, microwave irradiation significantly enhances biogas production from sludge (Yu et al. 2010). Subjecting WAS to microwave treatment at various temperatures results in a notable increase in both the rate and volume of biogas generated. The kinetic equation for biogas production from microwave-pretreated sludge is given in Eqs. 1–3 (Ebenezer et al. 2015; Ude and Oluka 2022), where *S* represents SCOD, *B* is the biogas yield, *R* is the reaction rate, *k* is the rate constant *Q*_*i*_ is the input flow rate, *Q*_0_ is the output flow rate, while *S*_*i*_ and *S*_0_ are the influent SCOD and effluent SCOD, respectively, and *V*_d_ is the digester volume. The improvement in biogas production can be attributed to the increased SCOD, as indicated by these equations.

1 $$\underbrace{SCOD}_S+Anaerobic\;Bacteria\xrightarrow{yileds}\;\underbrace{Biogas}_B$$

2 $$R= -k*S=k*B$$

3 $${V}_{d} \frac{dS}{dt}= {Q}_{i}*{S}_{i}-{Q}_{0}{S}_{0}+ {V}_{d}\left[-kS\right]$$

However, despite the benefits, energy consumption is crucial for scaling up microwave pretreatment technology for industrial application (Cano et al. 2015). Table 4 highlights the challenge of achieving a favourable energy balance, which is essential for the practical application of this technology on an industrial scale. Therefore, while microwave pretreatment offers significant potential in enhancing biogas production, optimizing the process to reduce energy consumption while maintaining high biogas yields is vital for its large-scale viability.

**Table 4:** Energy balance of microwave pretreatment in different sewage sludges

Pretreatment system	Pollution type	Energy input, *E*_i_ (kJ/kg VS)	Energy output, *E*_o_ (kJ/kg VS)	*E*_i_/*E*_o_ ratio	Ref
Microwave (60 ℃)	Food waste	50,000	738	68.8	(Yue et al. 2021)
Microwave (50 ℃)	Microalgae–bacterial biomass	36,700	525	70	(Passos et al. 2013)
Microwave (80 ℃)	Municipal sludge	114,000	5700	20	(Kor-Bicakci et al. 2019)
Microwave (40 ℃)	Municipal sludge	14,000	390	36	(Ebenezer et al. 2015a)
Microwave (40 ℃)	Municipal sludge	13,435	386	35	(Kavitha et al. 2016)
Microwave (80 ℃)	Municipal sludge	336,000	57,141	5.8	(Appels et al. 2013)
Microwave (70 ℃)	Municipal sludge	96,120	19,846	4.8	(Houtmeyers et al. 2014)
Microwave (150 ℃)	Wheat straw	279,400	2713	103	(Jackowiak et al. 2011)
Microwave (75 ℃)	Winery waste	142,069	13,488	10.5	(Pellera and Gidarakos 2017)

To enhance the energy viability of microwave pretreatment technology, significant research efforts have been made recently. Balasundaram et al. (2022) observed that low-temperature pretreatment (< 100 °C) reduces electrical energy consumption and achieves a positive energy balance, but the increased electrical demand from microwaves challenges the system’s energy self-sufficiency. Tang et al. (2010) highlighted that moisture content significantly affects microwave irradiation energy efficiency, and reducing sludge moisture content can decrease microwave energy input. Kavitha et al. (2018) implemented ultrasonic-assisted microwave pretreatment to increase methane yield, resulting in a net profit of US $2.67 per ton, although this study did not consider plant investment costs. Other studies, such as those by Kang et al. (2020), have utilized carbon nanotube-coated microwave vessels to enhance energy efficiency and sludge dissolution, showing promising results at the laboratory scale but lacking data from pilot and industrial-scale experiments. In conclusion, energy consumption remains a major barrier to the widespread adoption of microwave pretreatment, with the ongoing challenge being to reduce energy costs while increasing biogas production.

## Limitations and prospects

The effectiveness of microwave pretreatment in anaerobic sludge digestion, sludge dewatering, and increased biogas production has been established in multiple studies. Whether applied alone or in combination with chemical, physical, and other auxiliary methods, microwave pretreatment has shown great promise as a method for sludge pretreatment. Numerous laboratory-scale experiments provide ample evidence to support the application of this technology in larger-scale experimental studies.

Despite the maturity of microwave pretreatment for sludge effluent technology, the mechanistic study of microwave radiation remains controversial. The existence of microwave non-thermal effects and the modeling of microwave mechanisms for industrial-scale applications continue to be important directions for future development. While there have been some studies on the modeling of microwave heating, these predominantly focus on food (Campañone et al. 2012; Yang and Chen 2021), materials (Lovás et al. 2010; Goyal and Vlachos 2020), and chemistry (Zhang et al. 2000; Zhu et al. 2007). However, there remains a gap in the modeling of microwave treatment of sewage sludge. The integration of microwave heating with sludge structural analysis could significantly bolster the expansion of microwave pretreatment in industrial applications.

Industrial applications are another key factor limiting the wider use of microwave pretreatment. Most research in this area has focused on laboratory-scale (Zaker et al. 2019; Bozkurt and Apul 2020; Vialkova et al. 2021), although there have been pilot-scale (Kocbek et al. 2020; Guo et al. 2021) experiments confirming the potential successful application of microwave pretreatment technology. However, progress from laboratory to pilot scale has been hindered by apparent inconsistencies in performance in the original experiments. These inconsistencies can be attributed to factors such as variability in sludge characteristics and differences in microwave treatment parameters. For instance, Mawioo et al. (2017) used four diverse types of sludge (partially dewatered/centrifuged WAS, fresh faecal sludge, septic tank sludge, and WAS) to examine the performance of a microwave reactor at pilot scale. The results showed that microwave-based technology is a promising option for the treatment of faecal sludge, septic sludge, and WAS; however, due to differing organic contents, sanitization and volume reduction performances showed significant differences. Passos et al. (2015) compared thermal (95 °C, 10 h) and microwave irradiation (900 W, 3 min, 34.3 MJ/kg TS) for improving microalgae anaerobic digestion at pilot scale, finding that the best results were obtained with thermal pretreatment (95 °C, 10 h) rather than microwave irradiation, likely due to the choice of microwave treatment parameters.

Additionally, complexities in scaling up, such as managing uniform heating and precise parameter control in larger setups, equipment limitations for processing large volumes, and the economic challenges associated with larger-scale operations, contribute to these inconsistencies. Aguilar-Reynosa et al. (2017) point out that for the development of microwave reactors, it is necessary to further explore the design of applicators (traveling wave, multimode, and monomodal cavities) that enable high-power densities and faster heating rates, thereby assessing microwave heating processing as an alternative pretreatment in second-generation biorefineries.

Building upon these challenges, microwave pretreatment faces its own set of hurdles that need addressing to enable industrialization. Key among these is a more thorough understanding of the interactions and relationships between microwave radiation, biomass, and the heating medium, particularly concerning the non-thermal effects of microwave irradiation on biomass; the development of more efficient microwave absorbents that can effectively transfer energy to the biomass and be easily separated afterwards; and the upscaling of microwave pretreatment reactors to accommodate large-scale treatment of various types of sewage sludge.

## Conclusions

In conclusion, this scientometric analysis of 1763 bibliographic records provides a comprehensive overview of the development and current state of microwave pretreatment technology in sewage sludge treatment. The study traces the technology’s evolution, highlights significant advancements, identifies challenges, and suggests potential future directions. The global contributions and thematic focuses reflect the technology’s dynamic role in environmental management practices. The primary conclusions of this research are summarized as follows:

- Originating in the 1980s for trace substance measurement in sludge, microwave pretreatment technology has evolved significantly, becoming a key strategy for optimizing anaerobic digestion processes.
- Since 2010, there has been a notable shift in focus towards the mechanics of microwave pretreatment, aimed at enhancing energy efficiency and refining treatment techniques, indicating a progression to more advanced methodologies.
- Contributions from key figures like Cigdem Eskicioglu and research teams in China, India, and the USA highlight global interest. Research themes have centred around “microwave pyrolysis,” “anaerobic digestion,” “biogas,” and “degradation.”
- The study underscores a consistent emphasis on enhancing energy efficiency and exploring combinations with other treatment methods, signaling a mature phase in the development of microwave pretreatment methods.
- The transition from laboratory to industrial scale has faced challenges due to inconsistencies in performance and the need for comprehensive mathematical models for accurately representing microwave radiation mechanisms.
- A significant gap exists in the development of comprehensive models that capture the interactions between microwave radiation and sludge components, which is crucial for scalability and assessing environmental impact.
- Industrial-scale application of this technology confronts challenges in developing scalable operational models, evaluating economic viability, and understanding the comprehensive environmental impact.
- Fostering collaboration between academia and industry is essential for translating laboratory findings into practical, scalable solutions.
- Addressing the identified research gaps and focusing on practical applications are crucial for the transition of this technology from laboratory research to impactful industrial solutions, ensuring its sustainability and wider adoption.

## Data Availability

This literature review is based on publicly available sources, including peer-reviewed journal articles, conference papers, books, and online resources. Where possible, references have been directly linked to their source URLs or DOIs to facilitate access.
